# The Royal British Nurses' Association

**Published:** 1919-07-05

**Authors:** 


### The Royal British Nurses' Association.

THE Annual Meeting of the Royal British Nurses' Associations was held at the Medical Society's Rooms, Chandos Street, on Monday afternoon, June 30. Forty-five people were present. The chair was taken by Mr. H. J. Paterson, F.R.C.S. General reports were read by Miss Macdonald and by the Hon. Treasurer, Mr. Kenneth Stewart. Business was quickly disposed of. A brief allusion was made by Mr. H. J. Paterson to the Nurses' Registration Bill and to the "discreditable efforts" of the supporters of the College of Nursing to wreck that Bill—efforts which in time would surely recoil on their own heads. Dr. J. G. McGregor Robertson dealt with the proceedings occurring in the House of Commons, where "every conceivable misrepresentation and mis-statement was made by the supporters of the College Bill." (Shame.) Speaking himself to a member of the House of Commons upon the tactics employed by the College of Nursing, the advice given by the member was "to go and do the same." Mrs. Bedford Fenwick seconded these remarks, and added that nurses who joined the College signed away their own liberty. They could be thrown out of the College without a hearing, and whoever were responsible for such a clause were tyrants! The wrecking of their Bill was not such a set-back as appeared, for had they not the pledge of the new Minister of Health, Dr. Addison, to bring in a new Bill? That would be an enormous triumph, for from the Government they would get justice, and it was their duty to fight for justice and save their souls alive. The meeting thus ended with applause, and adjourned for tea.

The Annual Report states that the most important event of the past year has been the affiliation with the Chartered Corporation, of the Matrons' Council of Great Britain and Ireland, the Society for the State Registration of Trained Nurses, the National Union of Trained Nurses, the Scottish Nurses' Association, the Irish Nurses' Association, and the Fever Nurses' Association. Each of these Societies nominated a representative for election to the General Council of the Corporation. The Trained Nurses' Annuity Fund has been affiliated with the Helena Benevolent and Settlement Funds of the Corporation. The publication of the *Nurses' Journal*, which shows a net loss for the year of £36 10s. 1d., had been discontinued, and arrangements made for its insertion into the *British Journal of Nursing*. Seven medical men and 126 matrons and nurses were elected members and three resignations of membership received. The Hon. Treasurership of the Central Committee having fallen vacant, Mr. H. J. Paterson, F.R.C.S., was appointed to this office. The accounts of the General Fund of 1918 show an excess of income over expenditure of £212 19s. 3d. The amount received for registration fees was £20 4s. 6d. less than in the former year. This fall is accounted for by the decision to lower the amount from one guinea to five shillings, it being felt that the smaller sum would enable more nurses to make use of the Charter, and afford greater promise of success for the Bill for State Registration. The Hon. Treasurer in his financial report states that the year has been one of great crises to the nursing profession, and a continuance of increased expenditure must be expected if the efforts to promote State Registration and to improve the organisation of the profession are to have any measure of success.

